# Pathogenesis of Skin Carcinomas and a Stem Cell as Focal Origin

**DOI:** 10.3389/fmed.2018.00165

**Published:** 2018-05-29

**Authors:** Frank R. de Gruijl, Cornelis P. Tensen

**Affiliations:** Department of Dermatology, Leiden University Medical Center Leiden, Netherlands

**Keywords:** skin carcinoma, UV radiation, stem cells, quiescent, Lgr5, Lgr6

## Abstract

UV radiation in sunlight has long been recognized as the main exogenous cause of skin carcinomas. We present a brief historical perspective on the progress in understanding the pathogenesis of skin carcinomas, and recent advances. Sun-exposed skin carries numerous UV-related mutations, and skin carcinomas rank among the tumors with the highest mutational loads. In this multitude of mutations only a few are crucial in driving the tumor. Some are known from hereditary (skin) cancer syndromes and other recurrent ones have been validated in transgenic mice. Considering the continuous renewal of the epidermis, the question arises whether the lifelong residing stem cells are the main targets in skin carcinogenesis, a multistep process that would require ample time to evolve. Therefore, classic quiescent stem cells have been studied as potential tumor-initiating cells, as well as more recently discovered actively dividing stem cells (either Lgr5+ or Lgr6+). Interesting differences have emerged between experimental UV and two-stage chemical carcinogenesis, e.g., the latter appears to originate from follicular stem cells, in contrast to the former.

## Introduction

Skin cancers had already been linked to excessive sun exposure in the nineteenth century, specifically skin carcinomas were found predominantly in people with outdoor jobs. Genotoxicity, mutagenesis, and carcinogenesis by UV radiation, as present in sunlight, were experimentally established in the early decades of the twentieth century. Before the 2nd World War spectral analyses showed that DNA was the target of UV radiation for cell death and mutations (1, 2): i.e., well before Watson and Crick published the correct model of the structure of DNA, explaining how genes made up of DNA carried the genetic code which could be straightforwardly copied for daughter cells. Miscopies would introduce mutations. Consequently, replication of damaged DNA, hampering correct copying, for cell division was identified as the most prominent cause of mutagenesis. Carcinogenesis is considered to evolve primarily as a “multi-hit” process in which mutations accumulate in cells until a combination of mutations (and possibly other genetic defects and epigenetic modulatory effects) emerges which drive a cell to malignancy. As such a cell destined for malignancy requires time and cell divisions to transform, the most likely candidates would appear to be adult stem cells that constitute the very basis of tissue renewal. This premise was evidenced by a correlation that Tomasetti and Vogelstein (3) found between rate of stem cell division in various tissues and the risk of cancer. This led them to the controversial statement that most cancers are “bad luck” arising from an inherent risk of mutation in cell division. UV irradiation is known to cause epidermal hyperproliferation and hyperplasia. This would increase the UV-related risk of carcinomas originating from the epidermis (4), in addition to the risk derived from the genotoxicity of UV radiation.

## Human skin carcinomas and sun exposure

The skin is an evident frontier of the body in interactions with its environment. UV radiation in sunlight poses a recurrent (geno-) toxic challenge to skin, and like all life dwelling on the Earth's surface, it has powerful defense mechanisms, among which very importantly Nucleotide Excision Repair (NER) to maintain the integrity of the genome. A defect in NER increases the risk of skin cancer dramatically to the point that 50% of patients with Xeroderma pigmentosum succumb to multiple skin cancers before the age of 30 (5). NER eliminates the dominant UV-induced DNA damage (cyclobutane pyrimidine dimers, CPDs, and 6–4 photoproducts, 6–4 PPs) by a “cut-and-paste” action: cut out an oligo with the damage and fill in the gap using the complementary strand. As this UV-induced DNA damage occurs predominantly at neighboring pyrimidines in a DNA strand, the resulting mutations (mainly C > T) are located at dipyrimidine sites, and referred to as UV signature mutations. Strikingly, mutations in the *P53* tumorsuppressor gene of skin carcinomas show predominantly this UV signature (6). Microscopic clusters of cells (clones) overexpressing mutant P53 are present in chronically exposed skin, and presumed to be potential precursors of skin carcinomas (7). More recently, deep sequencing of 74 cancer-related genes (incl. *P53*) has shown that sun-exposed skin (from eye lid resections) is full of mutations (2–6/Mb), with a majority of UV signature mutations and an estimated average of 140 small clones/cm^2^ with a mutation in one of these 74 genes (8). Strikingly, another recent study found SCC-related mutations to be restricted to P53-overexpressing cell clusters (9).

The authors (8) noticed that the sun-exposed skin appeared clinically normal despite the high mutation load, and that the clones remained restricted in size. Apparently, the skin is inherently able to cope with a multitude of mutated clones. In experiments with Wnt-activated clones, it was shown that in signal exchange the normal cells were stimulated to outcompete the mutated cells (10). Much earlier, it was reported that low grade malignant keratinocytes were kept in check to contribute to epidermal homeostasis by surrounding normal keratinocytes (11). Hence, the outgrowth of cells into a tumor would appear to require the collapse of growth control by surrounding normal cells.

Considering the high mutation load in sun-exposed skin, it is no surprise that skin carcinomas belong to the absolute top of cancers with high mutation loads (10,000–100,000 per cell). Mutation load was found to be proportional to the immunogenicity of a tumor (12) and consequently proportional to the success of immunotherapy by check-point inhibition (13). In immunosuppressed organ transplant recipients the risk of skin cancer is raised, most dramatically the risk of squamous cell carcinoma, SCC (14) which correlated with preceding cutaneous HPV infections (15).

## Driver mutations

With an overwhelming load of mutations it would appear impossible to separate the driver mutations from passenger mutations. However, recurrent mutations within this multitude could be considered drivers, and earlier on, potential drivers were identified from syndromes with an inherited pre-disposition to develop cancers. A textbook example of the latter is the Gorlin syndrome (Basal Cell Nevus Syndrome, BCNS) where mutations in the tumorsuppressor *PTCH* gene predisposes to activation of the Hedgehog pathway (e.g., by loss of the wt allele by UV radiation) and subsequent formation of multiple basal cell carcinomas, BCCs (16). Also, most sporadic BCCs turned out to be driven by an activated Hedgehog pathway commonly involving mutations in *PTCH* or *SMO* (17). Activation of the Hedgehog pathway or ectopic expression of its downstream transcription factor, Gli1, in mouse skin gives rise to BCCs (18, 19).

In malignant progression of SCCs the RAS pathway was found to be activated (20, 21), however, apparently without any relevant recurrent mutations, notably rarely mutations in (*Ha-*)*RAS* genes (22). Next to a predominance of UV signature mutations in *P53*, nearly all SCCs were found to bear such mutations in one or more of the *NOTCH (1–4)* genes (23). *NOTCH1* mutations were already present in early stages of SCC development (24). Transgenic mice in which epidermal Notch signaling was blocked developed SCCs (25).

## What cell drives the outgrowth of human skin carcinoma?

It is notoriously difficult to propagate skin carcinoma cells *in vitro* and establish cell lines. Our group could only maintain fresh SCCs intact as explants (26). Others were successful in culturing SCC cells on fibroblasts (3T3) as feeder layers (27). In contrast to normal fibroblast, the cancer-associated fibroblasts (CAFs) appear to harbor a special class of fibroblasts facilitating invasion of SCC into the dermis (28). SCCs show a clear heterogeneity with differentiated keratinocytes (around keratin “pearls,” horny layer-like deposits) enclosed by germinative basal cell layers of keratinocytes bordering and infiltrating the stroma. Like in normal epidermis, the stem cells that drive SCC, the tumor-initiating cells (TICs), are logically expected to reside in the germinative compartment of the tumor. CD133 (prominin-1) is a tumor stem cell marker (e.g., in lung cancer), and not detectable in normal epidermal keratinocytes (proteinatlas.com). But some cells in germinative outer rim of SCCs are CD133-positive, about 1% of the tumor cells (27). Transferring as few as 100–1,000 of these CD133+ cells in combination with a million of human fibroblasts in matrigel into a pre-created subcutaneous space resulted in a 50% chance to spawn a new SCC in immune compromised mice (not capable of rejecting the human SCC). Evidently, the human SCC TICs needed the microenvironment of human fibroblast to support the outgrowth (generating appropriate CAFs?). It is not clear whether or how the CD133+ cells are related to stem cells of the normal human epidermis.

Similar results using the subcutaneous transplant assay have been obtained with CD200+ cells from BCCs, about 1–2% of the tumor cells (29). In contrast to CD133, CD200+ cells are present in normal skin: specifically in hair follicles in the region (the bulge) where stem cells reside in mice. However, this does not necessarily imply that the BCCs originate from these cells in hair follicles [although tracing mitochondrial DNA mutations by COX-deficiency would support this (30)]. Activation of the Hedgehog pathway and further transformation could conceivably lead to CD200 expression in the TICs. As monotherapies with SMO antagonists (e.g., vismodegib) inhibiting the Hedgehog pathway are not curative, the authors suggest to target the CD200+ cells instead for a permanent elimination of the tumor.

## Historical prelude to experimental skin carcinogenesis

Present day research on experimental skin carcinogenesis employs two basic mouse models, chemically or UV driven, which stem from historical observations on skin cancer in man. First of all, the surgeon Sir Percival Pott (a founder of orthopedics) reported in 1775 on the frequent occurrence of scrotal cancer (SCCs) among chimney sweeps in London, and recognized soot (coal tar) as the evident culprit (31). And secondly, skin carcinomas were linked to sun exposure at end of the nineteenth century. In Hamburg the dermatologist Unna (32) stated in his book on skin diseases that degenerative changes in the sun-exposed skin of sailors (“Seemanshaut”) were associated with skin carcinomas. In Bordeaux Dubreuilh (33) noticed that vineyard workers contracted remarkably more skin carcinomas than people living in the city. Further detailed observations on body locations of the carcinomas indicated that they were most likely caused by sunlight.

## Chemical carcinogenesis

Just before the First World War, the first experimental proof of tumor formation by coal tar was provided by the Japanese pathologist prof Yamagiwa. It was done in rabbits by repeated applications of coal tar to the ears. Yamagiwa had visited the Virchow Institute in Berlin where he learned about Virchow's irritation theory (“Reiztheorie”) of carcinogenesis (34). The experiment was modified in mice to include “cocarcinogens” (35), such as the “irritant” croton oil which “promoted” tumor outgrowth (reminiscent of the “Reiztheorie”). From these early experiments the standard classic two-stage protocol evolved in which a single genotoxic challenge with, for example, coal tar [or one of its ingredients like benzo(a)pyrene] irreversibly “initiated” tumors after which tumor development was “promoted” by a regimen of repeated applications of an “irritant” like croton oil (or its active ingredient 12-O-tetra- decanoyl-phorbol-13-acetate, TPA, activating PKC) (36). Tumor promotion was reversible in that tumors would not develop or regressed up on early termination of this regimen. This protocol yielded exophytically growing, wart-like, benign tumors (papillomas), and at a later stage some SCCs. *Ha-ras* mutations were commonly present in these tumors, notably already at the earliest tumor stages in hyperplastic foci in hair follicles (37). And even earlier, *Ha-ras* mutations could be detected by nested PCR from expanding clones in the in normal looking skin that had been subjected to the two-stage protocol (38). In contrast to *Ha-ras, p53* mutations occurred late in tumor progression and were linked to malignant conversion (39). Over a period of 80 years chemical carcinogenesis took central stage because of experimental convenience and because of its versatility in analysing the biology of carcinogenesis and in characterizing (anti-) carcinogenic substances and their interactions.

## UV carcinogenesis

Experimental proof of tumor induction by UV radiation was first published in 1928 by Findlay (40) who had chronically irradiated depilated albino mice for 8 months with a quartz mercury lamp. Interestingly, he also found that painting the animals with coal tar before irradiation speeded up the development of tumors (<3 months). Next, the prolific Brazilian professor of pathology Angel Roffo—who also pioneered in showing benzpyrene from tabacco to be carcinogenic—showed in the 1930s that the UV part in sunlight blocked by window glass (“UVB”) to be carcinogenic on rats (41, 42). The exact wavelength dependence (action spectrum) was determined much later in the 1990s for SCCs in hairless mice (43). The early experiments were done on the ears (and tails), or shaven backs of haired mice, but in the 1960s the more convenient and sensitive hairless mouse model was introduced which has become a standard in experimental UV carcinogenesis (44). In contrast to hairless mice, haired mice were reported to developed fibrosarcomas next to SCCs under chronic UV exposure (with substantially higher UV dosages than used on hairless mice). However, this was corrected by showing that the tumors were keratinocyte-derived (i.e., exclusively epidermal) and ranged from well differentiated to spindle cell carcinomas (45). The tumor progression in hairless mice was very similar to that in humans starting with endophytically growing actinic keratosis as benign precursor lesions (majority of tumors <2 mm across) of which a fraction progressed to malignant SCCs (majority > 3 mm) (44), and with a majority throughout bearing UV signature mutations in the tumorsuppressor *p53* (46); even before tumors appeared, microscopic clusters overexpressing mutant-p53 could be detected in the chronically sub-sunburn UV-exposed skin (47). *Ras* mutations were virtually lacking in the tumors: only 1 tumor with a *Ki-ras* mutation out of 32 tumors, none with a *Ha-ras* mutation (48). Only with a NER defect, in XPA mice, did *Ha-ras* mutations occur in UV-induced tumors which notably were benign papillomas as found in chemocarcinogenesis (49). The repair defect impaired removal of CPDs from the transcribed strand of *Ha-ras*. This introduced novel mutational targets for UV radiation corresponding with the oncogenic *Ha-ras* mutations. Overall, the mutational spectrum of UV SCCs in hairless mice resembled that of human SCCs, including Notch 1–4 mutations (50). In the 1970s it was discovered that UV-induced tumors were antigenic and that UV irradiation raised a specific immune tolerance toward these tumors (51). Recently, cutaneous papilloma virus infection was shown to enhance UV carcinogenesis (52). In all, experimental UV carcinogenesis shows striking parallels with human SCCs supporting the validity of the model.

## Stem cells

A remarkable difference between chemical and UV carcinogenesis appears to be the origin of the SCCs. After initiation, abrasion of the interfollicular (IF) epidermis did not affect development of chemo-SCCs, indicating that they originated from the hair follicles (53). In contrast, our group showed that apoptotic elimination of the IF basal layer by a single UV overdose nullified the UV carcinogenic regimen up to that point and carcinogenesis had to restart afterwards, indicating that UV-SCCs originated from the IF epidermis (54).

**Figure 1:**
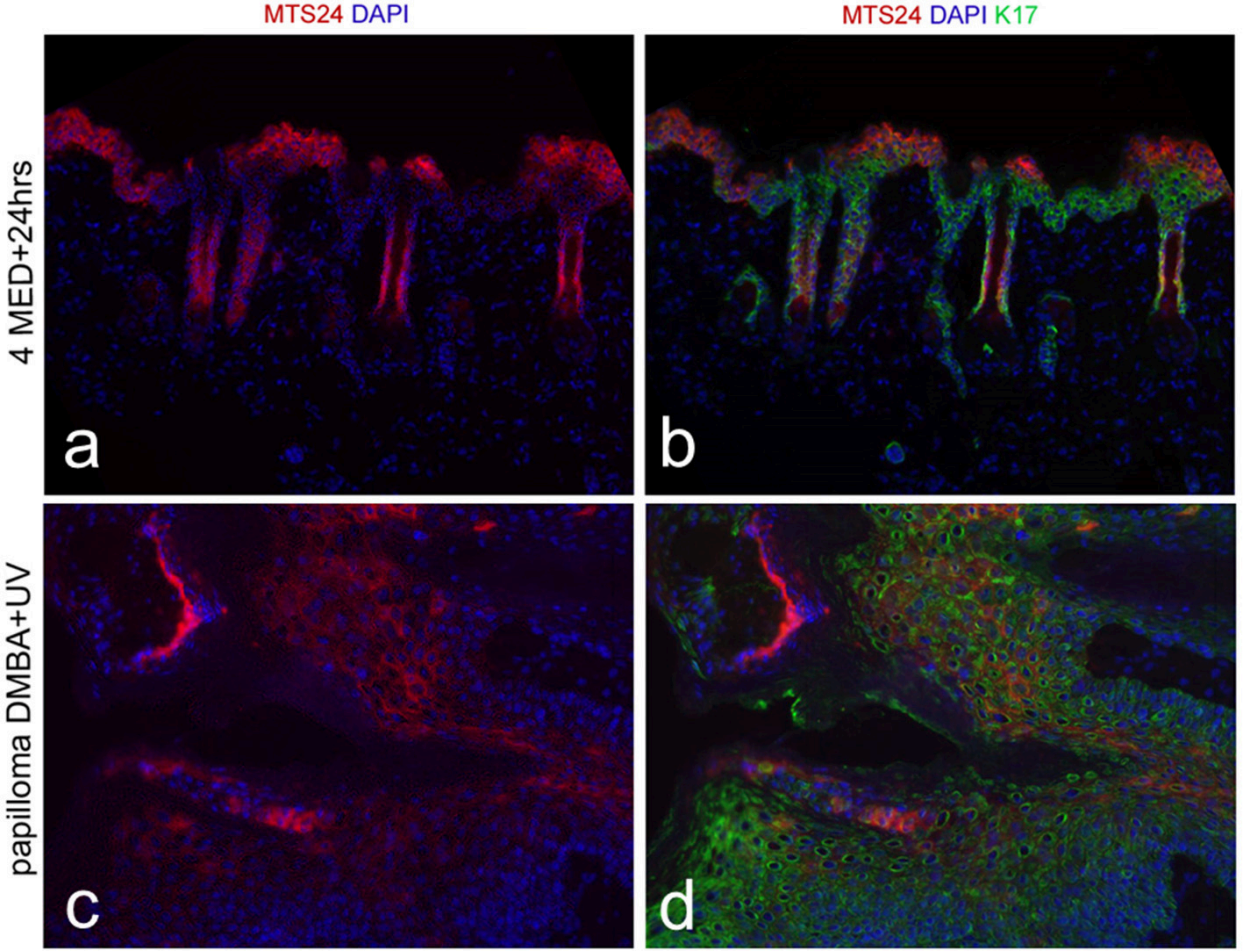
Mts24 fluorescence in red **(a,c)** in hairless mouse skin, combined with K17 in green **(b,d)**; **(a,b)** 24 h after high UV dose (4x threshold dose for a sunburn reaction) with Mts24+ cells high up in de epidermis in differentiated cell layers; **(c,d)** papilloma after neonatal DMBA (dimethylbenz [α]anthracene) followed by chronic UV exposure with Mts24+ cells throughout the tumor mostly differentiated cells (60), reproduced with permission.

The observation that the interval between tumor initiation and promotion could be extended to months demonstrated that the initiated cells were not shed in epidermal turnover and were therefore likely to be stem cells. This was confirmed by radioactive tracing of the initiating substance, benz(a)pyrene, which was retained in hair follicles and interfollicular epidermis in label-retaining cells, i.e., in quiescent stem cells (55). CD34+ cells located in the bulge of hair follicles were found to harbor such quiescent cells (56) and they were identified as tumor stem cells, or tumor-initiating cells (TICs), in chemically induced skin tumors (57). We similarly found that IF quiescent cells retaining CPDs from a low level UV regimen were linked to the development of non-regressing *in situ* carcinomas after TPA tumor promotion (58). There is, however, no established reliable protein marker for IF quiescent cells (resting or activated) by which to identify these cells in a tumor mass; putative stem cell markers (Wif-1, Lrig1, Dll1) did not label the CPD-retaining quiescent cells. Our group earlier identified Mts24/Plet1 as a stem cell marker (59) but later found this marker expressed in differentiated cells after UV exposure (Figures 1a,b) and in papillomas (Figures 1c,d) but absent in SCCs (not shown) (60). This demonstrated that a stem cell marker in homeostasis need not be one under (UV) stress, in hyperplasia or in tumors.

Recently, a new class of proliferating stem cells (either Lgr5+ or Lgr6+) was studied as possible TICs in chemical and UV carcinogenesis; this was done by “lineage tracing” to identify the progeny of these stem cells in tumors. Lgr5+ cells and progeny were not detected in either chemically or UV-induced tumors (61, 62). Our group could not detect any appreciable presence of Lgr6+ cells in tumors and only some sporadic remnants of progeny deep into the differentiated compartments, i.e., no indication that Lgr6+ cells were TICs or drove tumor growth (63). In contrast, Huang et al. (62) reported the presence of Lgr6+ cells in chemically induced tumors in a different mouse strain than we used, and with a different protocol for lineage tracing. However, these Lgr6+ cells did not exclusively reside in the germinative compartment of the tumors, showed a lack of expression of K14 (marker of germinative basal cells) and some even appeared flattened out in terminal differentiation. Apparently Lgr6 was no longer a marker of stem cells in these tumors (reminiscent of what we found with Mts24/Plet1). Intriguingly, Huang et al. (62) concluded from experiments with Lgr6 knockout mice that Lgr6 in normal epidermis functioned as a tumor suppressor. Interestingly in this respect, we found that Lgr6+ cells and progeny were lost from IF epidermis under chronic UV exposure long before the occurrence of SCCs; in contrast, a TPA regimen caused a clear expansion of progeny in the IF epidermis (63).

## Conclusion

From the present vantage point, UV carcinogenesis in mice appears to emulate SCCs in humans better than two-stage chemical carcinogenesis. And the quiescent stem cells appear to be the most likely target cells from which SCCs arise, either from quiescent cells in hair follicles in chemocarcinogenesis or quiescent cells in the IF epidermis in UV carcinogenesis. Future research should be directed toward identifying the latter cells by reliable protein markers, which may subsequently serve to develop well targeted interventions to prevent or cure cutaneous SCCs.

As there is no robust mouse model available for the *de novo* induction of BCCs by exogenous agents, identification of the primary target cells requires further research.

## Author contributions

FdG (biophysicist/photobiologist): outline of review, wrote draft, finalized text, and references. CT (molecular biologist): outline of review, brought in and checked molecular aspects, filled in some gaps, edited draft versions.

### Conflict of interest statement

The authors declare that the research was conducted in the absence of any commercial or financial relationships that could be construed as a potential conflict of interest.

## References

[B1] Hollaender A Emmons CW Wavelength dependence of mutation production in the ultraviolet with special emphasis on fungi. Cold Spring Harbor Symp Quand Biol. (1941) 9:179–86. 10.1101/SQB.1941.009.01.021

[B2] de Gruijl FR. Skin cancer and solar UV radiation. Eur J Cancer (1999) 35:2003–9. 10.1016/S0959-8049(99)00283-X 10711242

[B3] Tomasetti C Vogelstein B. Cancer etiology. Variation in cancer risk among tissues can be explained by the number of stem cell divisions. Science (2015) 347:78–81. 10.1126/science.1260825 25554788 PMC4446723

[B4] Berton TR Mitchell DL Fischer SM Locniskar MF. Epidermal proliferation but not quantity of DNA photodamage is correlated with UV-induced mouse skin carcinogenesis. J Invest Dermatol. (1997) 109:340–7. 10.1111/1523-1747.ep12335984 9284102

[B5] DiGiovanna JJ Kraemer KH. Shining a light on xeroderma pigmentosum. J Invest Dematol. (2012) 132:785–96. 10.1038/jid.2011.426 22217736 PMC3279615

[B6] Brash DE Rudolph JA Simon JA Lin A McKenna GJ Baden HP. A role for sunlight in skin cancer: UV-induced p53 mutations in squamous cell carcinoma. Proc Natl Acad Sci USA. (1991) 88:10124–8. 10.1073/pnas.88.22.10124 1946433 PMC52880

[B7] Jonason AS Kunala S Price GJ Restifo RJ Spinelli HM Persing JA. Frequent clones of p53-mutated keratinocytes in normal human skin. Proc Natl Acad Sci USA. (1996) 93:14025–9. 10.1073/pnas.93.24.14025 8943054 PMC19488

[B8] Martincorena I Roshan A Gerstung M Ellis P Van Loo P McLaren S. Tumor evolution. High burden and pervasive positive selection of somatic mutations in normal human skin. Science (2015) 348:880–6. 10.1126/science.aaa6806 25999502 PMC4471149

[B9] Albibas AA Rose-Zeerilli MJJ Lai C Pengelly RJ Lockett GA Theaker J Subclonal evoluation of cancer-related gene mutations in p53 immunopositive patches in human skin. J Invest Dermatol. (2018) 138:189–98. 10.1016/j.jid.2017.07.844 28844940

[B10] Brown S Pineda CM Xin T Boucher J Suozzi KC Park S. Correction of aberrant growth preserves tissue homeostasis. Nature (2017) 548:334–7. 10.1038/nature23304 28783732 PMC5675114

[B11] Vaccariello M Javaherian A Wang Y Fusenig NE Garlick JA. Cell interactions control the fate of malignant keratinocytes in an organotypic model of early neoplasia. J Invest Dermatol. (1999) 113:384–91. 10.1046/j.1523-1747.1999.00701.x 10469338

[B12] Schumacher TN Schreiber RD. Neoantigens in cancer immunotherapy. Science (2015) 348:69–74. 10.1126/science.aaa4971 25838375

[B13] Snyder A Makarov V Merghoub T Yuan J Zaretsky JM Desrichard A. Genetic basis for clinical response to CTLA-4 blockade in melanoma. N Engl J Med. (2014) 371:2189–99. 10.1056/NEJMoa1406498 25409260 PMC4315319

[B14] Madeleine MM Patel NS Plasmeijer EI Engels EA Bouwes Bavinck JN Toland AE. Epidemiology of keratinocyte carcinomas after organ transplantation. Br J Dermatol. (2017) 177:1208–16. 10.1111/bjd.15931 28994104

[B15] Genders RE Mazlom H Michel A Plasmeijer EI Quint KD Pawlita M. The presence of betapapillomavirus antibodies around transplantation predicts the development of keratinocyte carcinoma in organ transplant recipients: a cohort study. J Invest Dermatol. (2015) 135:1275–82. 10.1038/jid.2014.456 25347116

[B16] Athar M Li C Kim AL Spiegelman VS Bickers DR. Sonic hedgehog signaling in Basal cell nevus syndrome. Cancer Res. (2014) 74:4967–75. 10.1158/0008-5472.CAN-14-1666 25172843 PMC4167483

[B17] Pellegrini C Maturo MG Di Nardo L Ciciarelli V Gutiérrez García-Rodrigo C Fargnoli MC. Understanding the molecular genetics of basal cell carcinoma. Int J Mol Sci. (2017) 18:E2485. 10.3390/ijms18112485 29165358 PMC5713451

[B18] Oro AE Higgins KM Hu Z Bonifas JM Epstein EH Jr. Scott MP. Basal cell carcinomas in mice overexpressing sonic hedgehog. Science (1997) 276:817–21. 10.1126/science.276.5313.817 9115210

[B19] Nilsson M Undèn AB Krause D Malmqwist U Raza K Zaphiropoulos PG. Induction of basal cell carcinomas and trichoepitheliomas in mice overexpressing GLI-1. Proc Natl Acad Sci USA. (2000) 97:3438–43. 10.1073/pnas.97.7.3438 10725363 PMC16258

[B20] Einspahr JG Calvert V Alberts DS Curiel-Lewandrowski C Warneke J Krouse R. Functional protein pathway activation mapping of the progression of normal skin to squamous cell carcinoma. Cancer Prev Res. (2012) 5:403–13. 10.1158/1940-6207.CAPR-11-0427 22389437 PMC3297971

[B21] Hameetman L Commandeur S Bavinck JN Wisgerhof HC de Gruijl FR Willemze R. Molecular profiling of cutaneous squamous cell carcinomas and actinic keratoses from organ transplant recipients. BMC Cancer (2013) 13:58. 10.1186/1471-2407-13-58 23379751 PMC3570297

[B22] Mauerer A Herschberger E Dietmaier W Landthaler M Hafner C. Low incidence of EGFR and HRAS mutations in cutaneous squamous cellcarcinomas of a German cohort. Exp Dermatol. (2011) 20:848–50. 10.1111/j.1600-0625.2011.01334.x 21771097

[B23] Durinck S Ho C Wang NJ Liao W Jakkula LR Collisson EA. Temporal dissection of tumorigenesis in primary cancers. Cancer Discov. (2011) 1:137–43. 10.1158/2159-8290.CD-11-0028 21984974 PMC3187561

[B24] South AP Purdie KJ Watt SA Haldenby S den Breems N Dimon M. NOTCH1 mutations occur early during cutaneous squamous cell carcinogenesis. J Invest Dermatol. (2014) 134:2630–8. 10.1038/jid.2014.154 24662767 PMC4753672

[B25] Proweller A Tu L Lepore JJ Cheng L Lu MM Seykora J. Impaired notch signaling promotes de novo squamous cell carcinoma formation. Cancer Res. (2006) 66:7438–44. 10.1158/0008-5472.CAN-06-0793 16885339

[B26] Commandeur S de Gruijl FR Willemze R Tensen CP El Ghalbzouri A. An in vitro three-dimensional model of primary human cutaneous squamous cell carcinoma. Exp Dermatol. (2009) 18:849–56. 10.1111/j.1600-0625.2009.00856.x 19469895

[B27] Patel GK Yee CL Terunuma A Telford WG Voong N Yuspa SH. Identification and characterization of tumor-initiating cells in human primary cutaneous squamous cell carcinoma. J Invest Dermatol. (2012) 132:401–9. 10.1038/jid.2011.317 22011906 PMC3258300

[B28] Commandeur S Ho SH de Gruijl FR Willemze R Tensen CP El Ghalbzouri A. Functional characterization of cancer-associated fibroblasts of human cutaneous squamous cell carcinoma. Exp Dermatol. (2011) 20:737–42. 10.1111/j.1600-0625.2011.01305.x 21615509

[B29] Colmont CS Benketah A Reed SH Hawk NV Telford WG Ohyama M. CD200-expressing human basal cell carcinoma cells initiate tumor growth. Proc Natl Acad Sci USA. (2013) 110:1434–9. 10.1073/pnas.1211655110 23292936 PMC3557049

[B30] Fellous TG McDonald SA Burkert J Humphries A Islam S De-Alwis NM. A methodological approach to tracing cell lineage in human epithelial tissues. Stem Cells (2009) 27:1410–20. 10.1002/stem.67 19489031

[B31] Pott P Cancer scroti. In: Hawes L Clarke W Collins R editors. Chirurgical Observations Relative to the Cataract, the Polypus of the Nose, the Cancer of the Scrotum, the Different Kinds of Ruptures, and the Modification of the Toes and Feet. London: Longman (1775) p. 63–8.

[B32] Unna PG Die Histopathologie der Hautkrankheiten. Berlin: August Hirschwald Verlag (1894).

[B33] Dubreuilh H Des hyperkeratoses circumscriptes. Ann Derm Syph Paris (1896) 7:1158–204.

[B34] Fujiki H. Experimental study on the pathogenesis of epithelial tumors (I to VI reports). Cancer Sci. (2014) 105:143–9. 10.1111/cas.12333 24313817 PMC4317818

[B35] Berenblum I The mechanism of carcinogenesis. A study of the significance of cocarcinogenic action and related phenomena. Cancer Res. (1941) 1:44–8.

[B36] Balmain A Yuspa SH Milestones in skin carcinogenesis: the biology of multistage carcinogenesis. J Invest Dermatol. (2014) 134:E2–7. 10.1038/skinbio.2014.2 25302469

[B37] Binder RL Gallagher PM Johnson GR Stockman SL Smith BJ Sundberg JP. Evidence that initiated keratinocytes clonally expand into multiple existing hair follicles during papilloma histogenesis in SENCAR mouse skin. Mol Carcinog. (1997) 20:151–8. 10.1002/(SICI)1098-2744(199709)20:1<151::AID-MC17>3.0.CO;2-0 9328446

[B38] Finch JS Albino HE Bowden GT. Quantitation of early clonal expansion of two mutant 61st codon c-Ha-ras alleles in DMBA/TPA treated mouse skin by nested PCR/RFLP. Carcinogenesis (1996) 17:2551–7. 10.1093/carcin/17.12.2551 9006088

[B39] Kemp CJ. Multistep skin cancer in mice as a model to study the evolution of cancer cells. Semin Cancer Biol. (2005) 15:460–73. 10.1016/j.semcancer.2005.06.003 16039870

[B40] Findlay GM Ultraviolet light and Skin Cancer. Lancet (1928) 2:1070–3. 10.1016/S0140-6736(00)84845-X

[B41] Roffo AH Carcinomes et Sarcomes provoques par l'action du Soleil in toto. Bull Cancer Paris (1934) 23:590–616.

[B42] Roffo AH Üeber die physikalische aetiologie der krebskrankheit. Strahlenther (1939) 66:328–50.

[B43] de Gruijl FR Sterenborg HJ Forbes PD Davies RE Cole C Kelfkens G. Wavelength dependence of skin cancer induction by ultraviolet irradiation of albino hairless mice. Cancer Res. (1993) 53:53–60. 8416751

[B44] de Gruijl FR Forbes PD. UV-induced skin cancer in a hairless mouse model. Bioessays (1995) 17:651–60. 10.1002/bies.950170711 7646487

[B45] Morison WL Jerdan MS Hoover TL Farmer ER. UV radiation-induced tumors in haired mice: identification as squamous cell carcinomas. J Natl Cancer Inst. (1986) 77:1155–62. 2430133

[B46] Dumaz N van Kranen HJ de Vries A Berg RJ Wester PW van Kreijl CF. The role of UV-B light in skin carcinogenesis through the analysis of p53 mutations in squamous cell carcinomas of hairless mice. Carcinogenesis (1997) 18:897–904. 10.1093/carcin/18.5.897 9163673

[B47] Berg RJ van Kranen HJ Rebel HG de Vries A van Vloten WA Van Kreijl CF. Early p53 alterations in mouse skin carcinogenesis by UVB radiation: immunohistochemical detection of mutant p53 protein in clusters of preneoplastic epidermal cells. Proc Natl Acad Sci USA. (1996) 93:274–8. 10.1073/pnas.93.1.274 8552621 PMC40221

[B48] van Kranen HJ de Gruijl FR de Vries A Sontag Y Wester PW Senden HC. Frequent p53 alterations but low incidence of ras mutations in UV-B-induced skin tumors of hairless mice. Carcinogenesis (1995) 16:1141–7. 10.1093/carcin/16.5.1141 7767977

[B49] de Vries A Berg RJ Wijnhoven S Westerman A Wester PW van Kreijl CF. XPA-deficiency in hairless mice causes a shift in skin tumor types and mutational target genes after exposure to low doses of U.V.B. Oncogene (1998) 16:2205–12. 10.1038/sj.onc.1201744 9619829

[B50] Knatko EV Praslicka B Higgins M Evans A Purdie KJ Harwood CA. Whole-exome sequencing validates a preclinical mouse model for the prevention and treatment of cutaneous squamous cell carcinoma. Cancer Prev Res. (2017) 10:67–75. 10.1158/1940-6207.CAPR-16-0218 27923803 PMC5408961

[B51] Kripke ML Fisher MS. Immunologic parameters of ultraviolet carcinogenesis. J Natl Cancer Inst. (1976) 57:211–5. 10.1093/jnci/57.1.211 1003502

[B52] Hasche D Stephan S Braspenning-Wesch I Mikulec J Niebler M Gröne HJ. The interplay of UV and cutaneous papillomavirus infection in skin cancer development. PLoS Pathog. (2017) 13:e1006723. 10.1371/journal.ppat.1006723 29190285 PMC5708609

[B53] Morris RJ Tryson KA Wu KQ. Evidence that the epidermal targets of carcinogen action are found in the interfollicular epidermis of infundibulum as well as in the hair follicles. Cancer Res. (2000) 60:226–9. 10667563

[B54] Rebel HG Bodmann CA van de Glind GC de Gruijl FR. UV-induced ablation of the epidermal basal layer including p53-mutant clones resets UV carcinogenesis showing squamous cell carcinomas to originate from interfollicular epidermis. Carcinogenesis (2012) 33:714–20. 10.1093/carcin/bgs004 22227037

[B55] Morris RJ Fischer SM Slaga TJ. Evidence that a slowly cycling subpopulation of adult murine epidermal cells retains carcinogen. Cancer Res. (1986) 46:3061–6. 3698024

[B56] Trempus CS Morris RJ Bortner CD Cotsarelis G Faircloth RS Reece JM. Enrichment for living murine keratinocytes from the hair follicle bulge with the cell surface marker CD34. J Invest Dermatol. (2003) 120:501–11. 10.1046/j.1523-1747.2003.12088.x 12648211

[B57] Malanchi I Peinado H Kassen D Hussenet T Metzger D Chambon P. Cutaneous cancer stem cell maintenance is dependent on beta-catenin signalling. Nature (2008) 452:650–3. 10.1038/nature06835 18385740

[B58] van de Glind G Rebel H van Kempen M Tensen K de Gruijl F. Fractionation of a tumor-initiating UV dose introduces DNA damage-retaining cells in hairless mouse skin and renders subsequent TPA-promoted tumors non-regressing. Oncotarget (2016) 7:8067–77. 10.18632/oncotarget.6932 26797757 PMC4884976

[B59] Nijhof JG Braun KM Giangreco A van Pelt C Kawamoto H Boyd RL. The cell-surface marker MTS24 identifies a novel population of follicular keratinocytes with characteristics of progenitor cells. Development (2006) 133:3027–37. 10.1242/dev.02443 16818453

[B60] Nijhof JGW Stem Cells and Progenitor Cells as Targets in Skin Carcinogenesis. Ph.D. thesis, Leiden University, Leiden (2007).

[B61] van de Glind GC Out-Luiting JJ Rebel HG Tensen CP de Gruijl FR. Lgr5+ stem cells and their progeny in mouse epidermis under regimens of exogenous skin carcinogenesis, and their absence in ensuing skin tumors. Oncotarget. (2016) 7:52085–94. 10.18632/oncotarget.10475 27409834 PMC5239536

[B62] Huang PY Kandyba E Jabouille A Sjolund J Kumar A Halliwill K. Lgr6 is a stem cell marker in mouse skin squamous cell carcinoma. Nat Genet. (2017) 49:1624–32. 10.1038/ng.3957 28945253 PMC5662105

[B63] van de Glind GC Rebel HG Out-Luiting JJ Zoutman W Tensen CP de Gruijl FR. Lgr6+ stem cells and their progeny in mouse epidermis under regimens of exogenous skin carcinogenesis, and their absence in ensuing skin tumors. Oncotarget (2016) 7:86740–54. 10.18632/oncotarget.13436 27880932 PMC5349950

